# The characteristics of two LDL-cholesterol level reduction treatment strategies, “treat-to-target” and “percent reduction”: an observational study without intervention

**DOI:** 10.1186/s12872-019-1038-y

**Published:** 2019-03-11

**Authors:** Albert Császár

**Affiliations:** Military Hospital Department of Internal Medicine II, Hungarian Defense Forces Medical Center, H-1062 Podmaniczky u, Budapest, 111 Hungary

**Keywords:** Statin therapy, Percent LDL-cholesterol reduction, 2014 ACC/AHA guideline

## Abstract

**Background:**

The 2014 ACC/AHA guidelines redefined the strategy for LDL-cholesterol (LDL-C) treatment. According to data from evidence-based studies, the basis for earlier therapeutic recommendations for LDL-C target levels (2.6 and 1.8 mmol/L) may be disputed, and only the data for the percent LDL-C reduction are objective. The target is a moderate intensity (30–50%) LDL-C reduction in the high cardiovascular (CV) risk group, and a high intensity LDL-C reduction (> 50%) in the very high risk group. In our study, we analysed the success of the two types of strategies in attaining the target in the everyday routine.

**Methods:**

Of 5652 patients suffering from hypercholesterolemia, 4302 underwent treat-to-target treatment, and 1350 patients were treated with a percent reduction strategy. Physicians were free to choose the dosage and the target treatment form. The 12-month study included three follow-up visits.

**Results:**

In the high CV risk, statin-naive subgroup the percent LDL-C reduction strategy has been proven to be clearly more successful than the treat-to-target strategy, i.e. a higher proportion of patients reached the target values. We observed that the absolute value corresponding to a percent reduction target is higher if the baseline LDL-C is higher, and therefore it is easier to reach.

**Conclusion:**

Therefore, in this large subgroup of patients with baseline LDL-C level higher than 3.9 mmol/L may be recommended the adaptation of the percent reduction assessment.

## Background

The new US cholesterol treatment guideline (elaborated by the ACC/AHA societies) was first presented in 2013. The guideline recommends the administration of moderate- and high-intensity statin therapy, in contrast to the LDL-cholesterol (LDL-C) treatment targets used earlier [1]. The guideline clearly states that a detailed review of hard-endpoint major trials used as evidence offers no proof as to what specific absolute target should be used, as there was no evaluation of treatment targets, and no comparison of the attainment of the different treatment targets was done, nor were different dose strengths compared, except for three trials, which looked into this particular aspect. Based on the above, the document clearly regards statin therapy intensities as being useful on the basis of the available data, i.e. it defines a LDL-C level reduction of at least 50% for high-intensity, and a reduction of between 30 and 50% for moderate intensity. In other words, the recommendation is contrary to the earlier ATP III guidelines. The European guideline for the treatment of dyslipidaemia revised in 2016 (ESC/EAS societies) also accepts 50% reduction as an alternative [2]. The aim of our study was to compare the rate and the characteristics of success achievable with the two strategies for persons with high and very high CV risk.

## Methods

### Patients

The study was conducted by 583 family doctors and 101 hospital based specialists, who enrolled 5652 hypercholesterolemic patients (Table 1). The median age was 60.6 years, while the distribution by gender was 48% male and 52% female patients. Both the median age and the gender distribution were nearly identical in both treatment arms.

**Table 1:** Characteristics of patients

A. Data of Patients at high risk		Attained LDL-C Level	Percent LDL-C Reduction
Total N		3452	1130
Age (Years)	Mean	60,0	60,2
	Median	61	61
	Standard Error of Mean	0,18	0,30
Sex (%)	Male	45	48
	Female	55	52
Smoking (%)	Yes	45	47
	No	55	53
Hypertension (%)	Yes	78	75
	No	22	25
Type 2 Diabetes (%)	Yes	18	19
	No	82	81
Previous Treatment (%)	Naive, newly diagnosed	71	70
	Treated Patient not at goal	29	30
Total Cholesterol	Mean	6,4	6,6
	Median	6,5	6,6
	Standard Error of Mean	0,02	0,03
LDL	Mean	4,1	4,3
	Median	4,2	4,3
	Standard Error of Mean	0,02	0,04
HDL	Mean	1,5	1,5
	Median	1,3	1,3
	Standard Error of Mean	0,01	0,02
Trigyiceride	Mean	2,2	2,4
	Median	2,1	2,2
	Standard Error of Mean	0,02	0,03
B. Data of Patients at very high risk		Attained LDL-C Level	Percent LDL-C Reduction
Total N		850	220
Age (Years)	Mean	62,8	63,7
	Median	64	65
	Standard Error of Mean	0,31	0,66
Sex (%)	Male	55	57
	Female	45	43
Smoking (%)	Yes	68	67
	No	32	33
Hypertension (%)	Yes	84	86
	No	16	14
Type 2 Diabetes (%)	Yes	41	40
	No	59	60
Previous Treatment (%)	Naive, newly diagnosed	44	38
	Treated Patient not at goal	56	62
Total Cholesterol	Mean	6,1	6,2
	Median	6,1	6,2
	Standard Error of Mean	0,04	0,08
LDL	Mean	3,8	3,9
	Median	3,9	3,8
	Standard Error of Mean	0,04	0,09
HDL	Mean	1,4	1,5
	Median	1,2	1,2
	Standard Error of Mean	0,03	0,07
Trigyiceride	Mean	2,4	2,5
	Median	2,1	2,2
	Standard Error of Mean	0,05	0,10

Some of patients were newly diagnosed with hypercholesterolemia, and some had taken statins (all except rosuvastatin) previously, but not attained the desired target level. The risk classification was carried out based on the recommendations of the ESC/EAS guideline [2]. Four thousand three hundred two patients participated in the treat-to-target form of treatment, and 1350 patients in the percent reduction arm. The difference in the number of patients between two target strategies due to the fact that percent reduction judgement is a new, unexperienced model. In terms of risk stratification, the number of patients in the high-risk group without CV symptoms was considerably higher than the number of patients with a very high risk.

Treatment was done with rosuvastatin (Xeter®, Gedeon Richter Plc.), and we compiled a recommendation on the initial dose selection for both strategic arms based on our earlier experience relating to risk status and initial LDL-C levels. We took into consideration whether the patient had received statin therapy at some point previously, because the administration of larger doses is advised if this is the case.

Nevertheless, these were only suggestions, and there were no compulsory instructions regarding the choice of the initial dose or the dose change. The physicians made entirely independent decisions regarding the course of treatment (dosage and strategy). Our survey was an observational study without intervention.

During the twelve-month study, follow-up visits took place at 2 months and 6 months, followed by closing tests at month 12. During follow-up, ASAT and ALAT hepatic enzymes as well as CK and creatinine were also measured in addition to lipid levels, and we also registered the existence of any complaints that could be linked to taking the drug. The aim of the study was to establish the success rate of both treatment strategies. In the case of the treat-to-target strategy, success was defined as attaining an LDL-C level below 2.6 mmol/L in the high-risk population without CV symptoms, and below 1.8 mmol/L in the very high-risk group. In the case of percent reduction, success was defined as an LDL-C level reduction of between 30 and 50% in the high-risk group without CV symptoms, and a reduction of over 50% in the very high-risk group.

### Statistical analysis

SPSS module tables were used to compare the means, and a Bonferroni adjustment was performed with a t-test for comparison; the significance level was: Alpha: 0.05 For comparing distributions and proportions, SPSS table modules and a z-test with Bonferroni adjustment were used for comparison; significance level: Alpha: 0.05 In certain cases (e.g. liver function), a paired t-test was used.

## Results

**Fig. 1:**
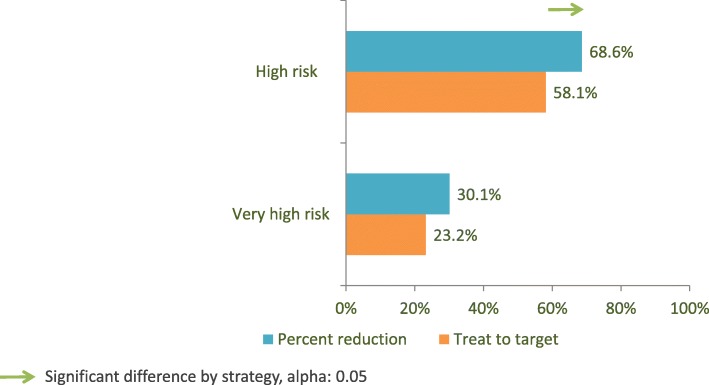
Rate of successful target attainment according to risk group

Taking risk strata into account, in the very high-risk group percent reduction had a 30.1% and treat-to-target a 23.2% success rate, respectively, which is not a significant difference. In the high-risk, no-CV symptoms patient cohort, percent reduction had a 68.6% success rate, while treat-to-target had 58.1%, a discrepancy which proved significant (Fig. 1). This means that the percent reduction strategy brings an advantage primarily to the high-risk patients.

**Fig. 2:**
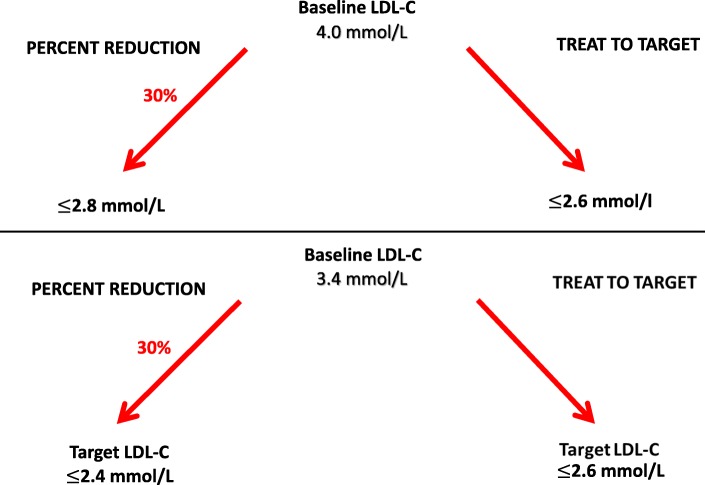
Target values in high risk patients with “percent reduction” or “treat to target” treatment strategies at different baseline LDL-C levels

The success rate of reaching the target depending on the initial LDL-C level was also analysed for both treatment strategies. We observed that below a limit value the higher the baseline LDL-C, the higher is the success rate of the percent reduction treatment. This is partly because with percent reduction, the higher the LDL-C level is, the higher the absolute value correspondences to the 30% reduction, and thus, that is more easily attained compared to the constant 2.6 mmol/L target level used in the treat-to-target approach. On the contrary, the lower the initial LDL-C data, the lower is the value which fits the 30% reduction rate and it can be dropped below 2,6 mmol/L, in that case treat to target model is more favourable. Figure 2 exposes the two discussed theoretical situations.

**Fig. 3:**
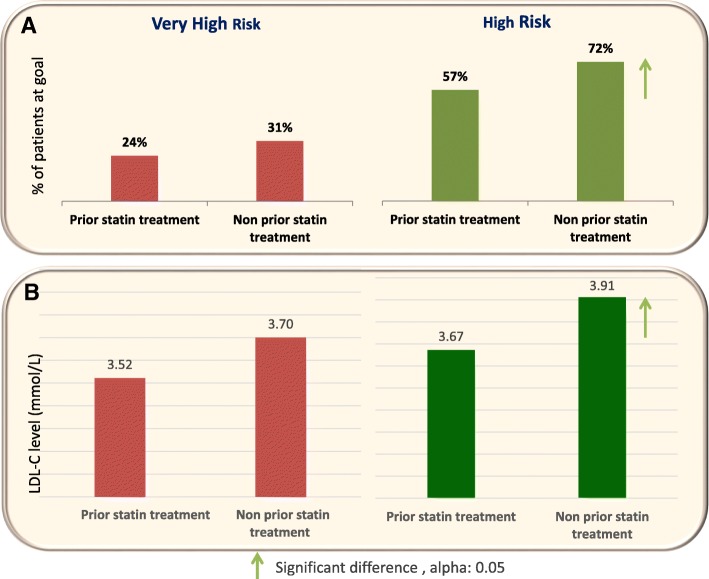
Proportions of patients with achieved goals (**a**) and baseline LDL-C levels. **b** by risk category and by prior statin treatment. Percent reduction strategy at month 12

Further in-depth analysis revealed that within the high-risk group the percent reduction strategy had a better achievement with the statin-naive patients (Fig. 3a), clarified by the fact that these patients had higher baseline LDL-C values (3.9 mmol/L) than the previously statin-treated patients who evidently had lower baseline values (3.6 mmol/L) (Fig. 3b). The same also applies for the very high-risk statin-naive patients, whose baseline LDL-C values are likewise higher, although in this case, the difference between the proportions of patients reaching the target value is not statistically significant (Fig. 3). These findings supported de facto our hypothesis (Fig. 2).

When analysing the rosuvastatin doses, the question arises as to whether the higher success rate in the percent reduction group can or cannot be explained by the higher doses. The analysis showed that at month 12 there was no difference in the proportion of patients receiving the 10 mg dose (25% vs 23%). There were fewer patients taking the 20 mg dose in the treat-to-target arm (58% vs 64%); nevertheless, the use of the 40 mg dose was more common in this treatment strategy arm (16% vs 12%), which means that the proportions by themselves do not give any explanation. There were also no significant differences according to age and sex.

Theoretically, the disease-modifying effect of the non-statin lipid-lowering agents could also be taken into consideration. Our findings show, however, that the use ratio of such agents was low in both arms and did not differ much (3.8% vs 3.3%).

## Discussion

One of the most remarkable findings related to the importance of LDL-C percent reduction was made in the REVERSAL study [3]. Head-to-head comparison of pravastatin 40 mg and atorvastatin 80 mg was performed, and IVUS was used to assess the plaques. It was concluded that, in the case of atorvastatin, plaque regression occurred if the initial LDL-C level was reduced by at least 50%. The data was later included in several guidelines, including the previous ESC/EAS recommendation.

Far more important in this respect is the 2014 ACC/AHA guideline [1], during the elaboration of which it was found that evidence could only be established between LDL-C percent reduction and percent reduction of simultaneous CV events. The RCTs conducted so far did not specify treatment targets nor did they compare any treatment target values.

The dyslipidaemia treatment guideline by the British NICE institute in 2014 [4] also emphasizes that a maximum statin dose (e.g. atorvastatin 80 mg) should be used in the case of patients with known CV disease, and a non-HDL cholesterol reduction of at least 40% should be achieved. Thus, this guideline also abandons specific target levels.

According to the latest ESC/EAS guideline (2016), at least a 50% LDL-C reduction is recommended as an alternative in the case of high-risk and very high-risk patients [2].

A recently published study compared retrospectively the significance of percent reduction and treat-to-target from the perspective of preventing CV events [5] using data from 3 large statin trials (TNT, IDEAL, SPARCL). Patients were grouped based on the LDL-C target level (< 1.8 vs. > 1.8 mmol/L) and LDL-C percent reduction (< 50% vs. > 50%). MACE was the primary endpoint, and the associated prognostic value was compared using the Cox proportional hazards model. According to the study based on data collected from nearly 14,000 patients, the importance of LDL-C percent reduction was significantly higher than the role of treating to a desired LDL-C level. This is suggested by the outcomes seen in patients with therapeutic LDL-C levels < 1.8 mmol/L, reaching or not reaching a 50% LDL-C reduction. Additional CV benefits clearly occurred in those reaching or exceeding the 50% reduction rate. Conversely, patients reaching or exceeding the 50% reduction rate did not experience any further benefits even if they reached the 1.8 mmol/L target.

The main objective of our study, i.e. the proportion of patients reaching the target values, was significantly different in the 2 treatment strategy arms only in the high-risk group. Additional analysis showed that the percent reduction had a higher success rate only in the statin-naive patients whose baseline LDL-C levels was much higher. The very high-risk patients who were treated with statins previously had lower baseline LDL-C values; however, at these levels the higher success rate of the percent reduction therapy didn’t realize.

Nevertheless, the study has some limitations. This investigation is an observational study without intervention and ranks well below randomized study. The number of patients in the two strategy arms is imbalanced and this the proportion reflects the fact that percent reduction pattern is a new version with few practical experience. However, the great number of patients in two arms may merit statistical power.

## Conclusion

The authors would like to stress the benefits with the use of the percent reduction manage which had a higher success rate in the high-risk, statin-naive patients with LDL-C level 3.9 mmol/l or higher and represents a powerful alternative approach to reach target.

## Abbreviations

ACC/AHA: American College of Cardiology/American Heart Association
ALAT: Alanine aminotransferase
ASAT: Aspartate transaminase
ATP: Adult Treatment Panel
CV: Cardiovascular
ESC/EAS: European Society of Cardiology /European Atherosclerosis Society
IVUS: Intravascular ultrasound
LDL-C: Low-density lipoprotein cholesterol
MACE: Major Adverse Cardiovascular Event
RCT: Randomized controlled trial
